# Use of the PsycheMERGE Network to Investigate the Association Between Depression Polygenic Scores and White Blood Cell Count

**DOI:** 10.1001/jamapsychiatry.2021.2959

**Published:** 2021-10-20

**Authors:** Julia M. Sealock, Younga H. Lee, Arden Moscati, Sanan Venkatesh, Georgios Voloudakis, Peter Straub, Kritika Singh, Yen-Chen A. Feng, Tian Ge, Panos Roussos, Jordan W. Smoller, Guanhua Chen, Lea K. Davis

**Affiliations:** 1 Division of Genetic Medicine, Department of Medicine, Vanderbilt University Medical Center, Nashville, Tennessee; 2 Vanderbilt Genetics Institute, Vanderbilt University Medical Center, Nashville, Tennessee; 3 Psychiatric & Neurodevelopmental Genetics Unit, Center for Genomic Medicine, Massachusetts General Hospital, Boston; 4 Center for Precision Psychiatry, Department of Psychiatry, Massachusetts General Hospital, Boston; 5 Stanley Center for Psychiatric Research, Broad Institute of Harvard and MIT, Cambridge, Massachusetts; 6 The Charles Bronfman Institute for Personalized Medicine, Icahn School of Medicine at Mount Sinai, New York, New York; 7 Department of Genetics and Genomic Sciences, Icahn School of Medicine at Mount Sinai, New York, New York; 8 Pamela Sklar Division of Psychiatric Genomics, Icahn School of Medicine at Mount Sinai, New York, New York; 9 Friedman Brain Institute, Icahn School of Medicine at Mount Sinai, New York, New York; 10 Department of Psychiatry, Icahn School of Medicine at Mount Sinai, New York, New York; 11 Icahn Institute for Data Science and Genomic Technology, Icahn School of Medicine at Mount Sinai, New York, New York; 12 Mental Illness Research, Education and Clinical Centers, James J. Peters VA Medical Center, Bronx, New York; 13 Department of Biostatistics and Medical Informatics, University of Wisconsin, Madison; 14 Department of Molecular Physiology and Biophysics, Vanderbilt University Medical Center, Nashville, Tennessee; 15 Department of Psychiatry and Behavioral Sciences, Vanderbilt University Medical Center, Nashville, Tennessee; 16 Department of Medicine, Vanderbilt University Medical Center, Nashville, Tennessee; 17 Department of Biomedical Informatics, Vanderbilt University Medical Center, Nashville, Tennessee

## Abstract

**Importance:**

Although depression is a common psychiatric disorder, its underlying biological basis remains poorly understood. Pairing depression polygenic scores with the results of clinical laboratory tests can reveal biological processes involved in depression etiology and in the physiological changes resulting from depression.

**Objective:**

To characterize the association between depression polygenic scores and an inflammatory biomarker, ie, white blood cell count.

**Design, Setting, and Participants:**

This genetic association study was conducted from May 19, 2019, to June 5, 2021, using electronic health record data from 382 452 patients across 4 health care systems. Analyses were conducted separately in each health care system and meta-analyzed across all systems. Primary analyses were conducted in Vanderbilt University Medical Center’s biobank. Replication analyses were conducted across 3 other PsycheMERGE sites: Icahn School of Medicine at Mount Sinai, Mass General Brigham, and the Million Veteran Program. All patients with available genetic data and recorded white blood cell count measurements were included in the analyses. Primary analyses were conducted in individuals of European descent and then repeated in a population of individuals of African descent.

**Exposures:**

Depression polygenic scores.

**Main Outcomes and Measures:**

White blood cell count.

**Results:**

Across the 4 PsycheMERGE sites, there were 382 452 total participants of European ancestry (18.7% female; median age, 57.9 years) and 12 383 participants of African ancestry (61.1% female; median age, 39.0 [range, birth-90.0 years]). A laboratory-wide association scan revealed a robust association between depression polygenic scores and white blood cell count (β, 0.03; SE, 0.004; *P* = 1.07 × 10^−17^), which was replicated in a meta-analysis across the 4 health care systems (β, 0.03; SE, 0.002; *P* = 1.03 × 10^−136^). Mediation analyses suggested a bidirectional association, with white blood cell count accounting for 2.5% of the association of depression polygenic score with depression diagnosis (95% CI, 2.2%-20.8%; *P* = 2.84 × 10^−70^) and depression diagnosis accounting for 9.8% of the association of depression polygenic score with white blood cell count (95% CI, 8.4%-11.1%; *P* = 1.78 × 10^−44^). Mendelian randomization provided additional support for an association between increased white blood count and depression risk, but depression modeled as the exposure showed no evidence of an influence on white blood cell counts.

**Conclusions and Relevance:**

This genetic association study found that increased depression polygenic scores were associated with increased white blood cell count, and suggests that this association may be bidirectional. These findings highlight the potential importance of the immune system in the etiology of depression and may motivate future development of clinical biomarkers and targeted treatment options for depression.

## Key Points

**Question:**

Are genes that increase predisposition to depression associated with increased inflammatory biomarkers, specifically white blood cell count?

**Findings:**

In this genetic association study of 382 485 participants, an association was noted between depression polygenic scores and white blood cell count across 4 independent biobanks. Mediation analyses suggest a bidirectional association between white blood cell count and depression diagnosis and implicate neutrophils as the main driver of the association.

**Meaning:**

These findings suggest that genes associated with depression (rather than only the clinical presentation of depressive symptoms) may be implicated in the proinflammatory state observed in clinical depression; this outcome may motivate future development of targeted biomarker panels and treatments.

## Teaser

This genetic association study uses data from Vanderbilt University Medical Center and the PsycheMERGE network to investigate the association between inflammatory biomarkers, such as white blood cell count, and the genes that predispose an individual to depression.

## Introduction

Depression is a common psychiatric disorder estimated to affect 264 million individuals worldwide.^1^ Diagnostic criteria for depression include clinical evaluation of self-reported psychiatric symptoms, such as depressed mood, irritability, anhedonia, or suicidal thoughts. In addition to psychiatric effects, depression is associated with increased risk for cardiovascular disease,^2,3,4^ autoimmune disease,^5^ and diabetes.^6,7,8,9^ The increased risk of peripheral diseases suggests the biology of depression is not limited to the brain; nevertheless, the causes and biological effects of depression in the brain and the periphery remain poorly understood.

In a health care setting, laboratory tests aid clinicians in diagnostic and treatment decision-making. Tests that can accurately and reproducibly indicate a medical state are generally referred to as biomarkers.^10^ To date, there are no biomarkers for depression; however, consistent with the high number of common comorbidities, depression is associated with changes in a wide range of clinical laboratory values, including increased proinflammatory cytokines,^11,12,13,14^ altered lipids,^15,16,17^ growth factors,^18,19,20^ and decreased brain-derived neurotrophic factor.^21,22,23,24^ For many of these physiological quantitative values, the underlying biological mechanisms are well understood. Further understanding of the biological link between clinical depression and these laboratory values can help identify the biological processes contributing to depression and could lead to the development of more informative biomarker panels to be used in risk assessment and treatment response.

Previous studies report a bidirectional association between depression and autoimmune disease.^25^ Several immune biomarkers are increased in patients with depression compared with controls, including monocytes,^26,27,28,29,30,31^ neutrophil-lymphocyte ratio,^14,32,33^ and C-reactive protein.^14,31,34,35^ However, most immune biomarker studies of depression are limited in sample size and scope and are often unable to control for potential confounders or determine the pathway between depression and biomarkers.

Electronic health records (EHRs) store longitudinal information about the health and clinical care of individual patients. Biobanks that link EHRs to DNA information provide an opportunity to analyze clinical information along with genetic risk factors. Genetic risk for depression can be estimated using polygenic scores (PGS), which aggregate the small effects of thousands of loci across the genome into 1 score for each individual.^36^ Although they are not currently recommended for clinical use, PGS do capture a significant proportion of the variance in depression diagnosis (1.5%-3.2%^37^), indicating that PGS represent a biologically relevant contribution to depression. In this work, we use recently developed methods^38^ to combine depression PGS with laboratory results stored in EHRs to robustly identify physiological processes affected by increased genetic liability to depression.

Whereas independent biobanks can be used to discover associations, combining multiple health record systems through consortia can validate those discoveries in broader populations. The PsycheMERGE Network consists of investigators from institutions across the US with the common goal of using EHRs and biobanks to advance the identification, biology, and treatment of psychiatric disorders.^39^ Here, we investigate the effect of polygenic risk for depression on clinically measured laboratory values leveraging data from 4 health care systems participating in the PsycheMERGE Network.

## Methods

### Sample Description

Electronic health record and genotype information was extracted for individuals of European descent across 4 biobanks in the PsycheMERGE Network: Vanderbilt University Medical Center (VUMC), Mass General Brigham (MGB), Million Veteran Program (MVP), and Mount Sinai Icahn School of Medicine (MSSM). Biobank-specific information can be found in the eMethods in Supplement 1. All participants provided informed consent, and study procedures were approved by each institution’s respective institutional review board. This genetic association study was conducted from May 19, 2019, to June 5, 2021, and followed the Strengthening the Reporting of Genetic Association Studies (STREGA) reporting guideline.

### Statistical Analyses

#### Depression Polygenic Scoring

Depression PGS were generated using Polygenic Risk Score–Continuous Shrinkage^40^ with single nucleotide variation (SNV) weights from the largest available depression meta-analysis.^37^ The linkage disequilibrium reference panel was constructed from 503 European samples in the 1000 Genomes Project phase 3.^41^ Polygenic scores were scaled to have a mean of 0 and a unit SD so that effect size estimates in subsequent analyses were interpreted per 1 SD increase in depression PGS. In VUMC data, the depression PGS explained 0.8% of the variance in major depressive disorder diagnosis (*P* = 3.85 × 10^−55^).

#### Laboratory-wide Association Scan of Depression PGS in VUMC

At VUMC, all laboratory results were extracted from the EHRs of 72 634 individuals of primarily European ancestry and 12 383 individuals of primarily African ancestry and cleaned as previously described^38^ (eMethods in Supplement 1). Associations between the depression PGS and laboratory results were estimated with a laboratory-wide association scan (LabWAS) approach^38^ controlled for sex and the top 10 genetic principal components (eTable 1 in Supplement 2). In conditional analyses, the LabWAS of depression PGS was covaried for body mass index (median across each individual’s EHR) and for depression (eTable 2 in Supplement 3), anxiety (eTable 3 in Supplement 4), adjustment reaction (eTable 4 in Supplement 5 and eTable 5 in Supplement 6), and tobacco use disorder (as a proxy for smoking status) (eTable 6 in Supplement 7) diagnoses, defined by phecodes 296.2, 300.1, 304, and 318, respectively (eFigure 1 in Supplement 1). In sensitivity analyses focused on smoking, we obtained smoking data from the social history forms within the EHR and extracted an ever/never smoking variable, which indicates whether an individual has ever smoked. We tested the ever/never smoking variable as a covariate in the LabWAS of depression PGS (eFigure 1 in Supplement 1; eTable 7 in Supplement 8). Genetic analyses must be conducted in ancestry-stratified samples to avoid confounding due to population stratification. Using principal component analysis, we determined the genetic ancestry of individuals in our sample.^38^ The largest continental ancestry group was European; therefore, we restricted the study participants to individuals of European ancestry in our primary analyses. The second largest ancestry was African. We conducted an initial LabWAS in the African sample; however, due to the small sample size, there were no significant associations. As a result, we did not continue with the sensitivity analyses in this sample. Primary analyses were restricted to individuals of European descent and repeated in individuals of African ancestry (n = 12 383) in VUMC only (eFigure 4 in Supplement 1; eTable 8 in Supplement 9).

#### Sensitivity Analyses in VUMC

A series of conditional and sensitivity analyses were performed to ensure the association between depression PGS and white blood cell (WBC) count was not due to a common comorbid confounder phenotype present in individuals with both an increased depression PGS and an increased WBC count. To find phenotypes associated with both depression PGS and WBC count, separate phenome-wide association scans were conducted of depression PGS and of the median, age-adjusted, inverse normal transformed WBC measurement (eMethods in Supplement 1). Next, phenotypes that were significantly associated with both depression PGS and WBC count at Bonferroni significance (WBC *P* < 3.64 × 10^−5^, depression PGS *P* < 3.72 × 10^−5^) were selected and binned into 7 categories based on phenotypic similarity. Group-based case-control variables were constructed, in which an individual was considered a case if they were a case for any of the group’s phecodes. Controls were required to be a control for all phecodes. To assess the effect of the comorbid phenotypes on the association between depression PGS and WBC, a series of linear regression analyses were conducted controlling for each of the groups separately and all common phenotype groups together. All analyses were controlled for sex, top 10 genetic principal components, and median age across the medical record. We also conducted a series of sensitivity analyses controlling for the impact of WBC genetics on the association between depression PGS and WBC count. Details can be found in the eResults, eFigure 5, and eTable 16 in Supplement 1.

#### Replication in the PsycheMERGE Network

Targeted replication analyses focused on depression PGS and WBC count were conducted in 3 external biobanks. Depression PGS were constructed and WBC quality was controlled for as in the VUMC biobank. The depression PGS and WBC counts were fitted in a linear regression model controlling for sex and top 10 genetic principal components. The associations controlling for depression and anxiety diagnoses were also replicated using the same phenotype definition as described in the discovery LabWAS at VUMC. The effect estimates from each analysis were meta-analyzed across all 4 sites using a fixed-effect inverse variance weighted model in the meta^42^ R package (R Core Team).

#### Depression PGS and WBC Mediation Analysis

Two mediation models were investigated using the mediation^43^ R package. First, WBC count was modeled as the mediator between depression PGS (exposure) and depression diagnosis (outcome). Second, depression diagnosis was modeled as the mediator between depression PGS (exposure) and WBC count (outcome). Details can be found in the eMethods in Supplement 1. The proportion-mediated estimates from all 4 sites were meta-analyzed using a fixed-effect inverse variance weighted model in the meta^42^ R package. Owing to the uniqueness of MVP (ie, combat exposed, primarily men) compared with the other sites, we also conducted meta-analyses excluding MVP (eTables 12 and 13 in Supplement 1).

#### Depression PGS and WBC-Differential Mediation Analysis

To determine which WBC subtypes contributed to the association between depression PGS and depression diagnosis, a series of multiple mediator analyses were conducted using the mediation^43^ R package. Each WBC subtype was analyzed as the main mediator between depression PGS (exposure) and depression diagnosis (outcome) with the remaining subtypes as the alternative mediators. Details can be found in the eMethods in Supplement 1.

#### Mendelian Randomization

We conducted bidirectional mendelian randomization between depression and WBC count using generalized summary-based mendelian randomization ^44^ in the GCTA, or Genome-wide Complex Trait Analysis, package, version 1.92.4.^45^ Index SNVs were selected using the default settings in GCTA: *P* value threshold of 5 × 10^−8^, linkage disequilibrium *r^2^* clumping threshold of 0.05, and a HEIDI-outlier threshold of 0.01 to remove SNVs that have pleiotropic effects on both risk factor and disease. From the depression^37^ and WBC^46^ summary statistics, 47 and 203 SNVs were selected as index SNVs, respectively.

Phenome-wide association scans, LabWAS, and conditional, replication, and mediation analyses were conducted using R, version 3.4.3 (R Foundation). Mendelian randomization was conducted using GCTA. The code for each analysis can be found online.^47^

## Results

### LabWAS of Depression PGS 

Across the 4 PsycheMERGE sites, there were 382 452 participants of European ancestry (18.7% female and 81.3% male; median age, 57.9 years). An additional 12 383 participants of primarily African ancestry (61.1% female; median age, 39.0 [range, birth-90.0 years]) were included from VUMC.

**Figure 1.**
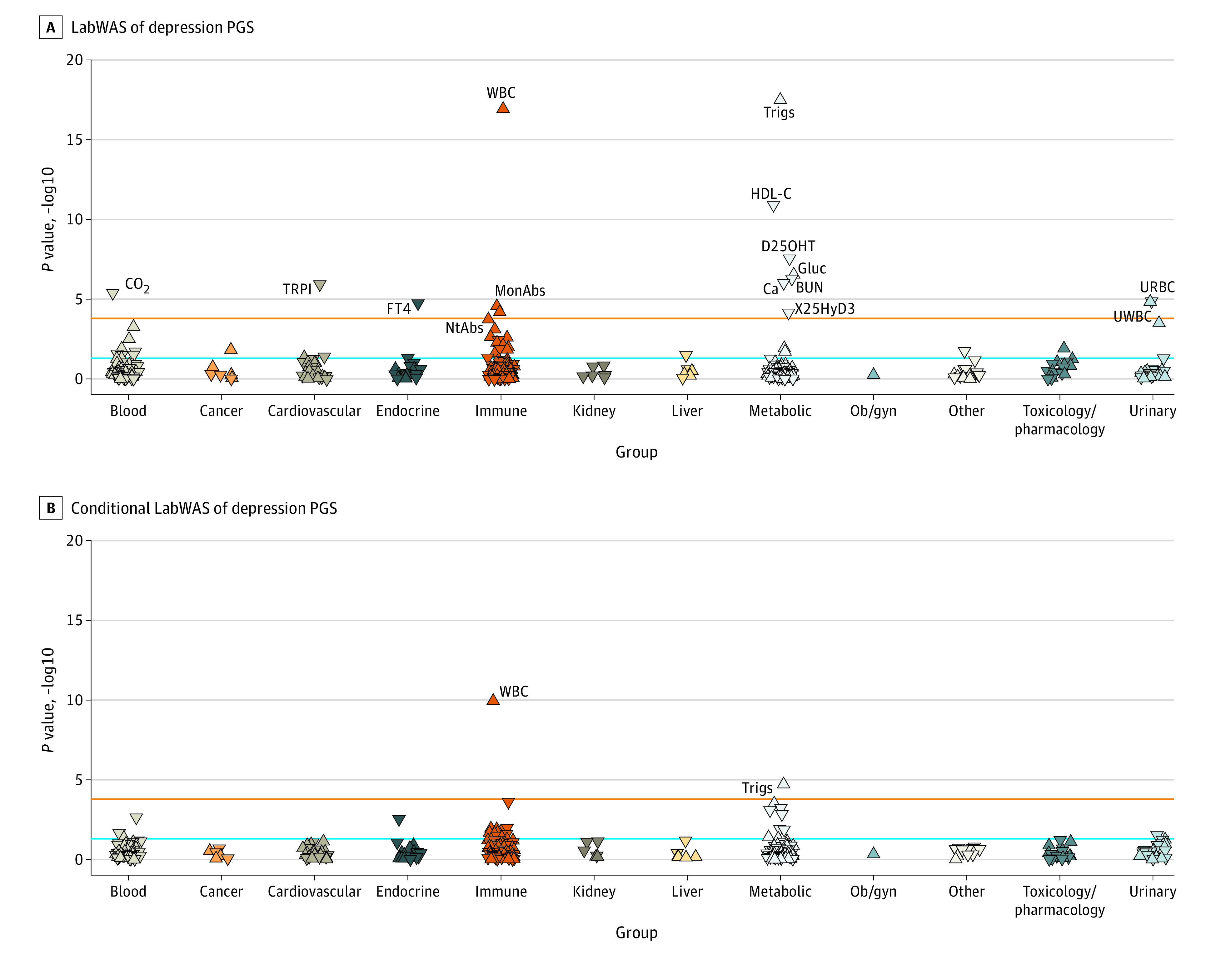
Laboratory-wide Association Scan (LabWAS) of Depression Polygenic Scores (PGS) Results of the initial LabWAS of depression PGS in Vanderbilt University Medical Center (A), and the LabWAS controlling for diagnoses for depression, anxiety, adjustment reaction, tobacco use disorder, and median body mass index across the electronic health record (B). The orange line indicates Bonferroni significance (*P* < 1.58 × 10^−4^), and the blue line represents *P* = .05. The direction of the arrow indicates the direction of effect between the laboratory and depression PGS. BUN indicates blood urea nitrogen; Ca, calcium; CO_2_, carbon dioxide; D25OHT, 25-hydroxyvitamin D; FT4, free thyroxine; Gluc, glucose; HDL-C, high-density lipoprotein cholesterol; MonAbs, absolute monocyte count; NtAbs, absolute neutrophil count; ob/gyn, obstetrics and gynecology; Trigs, triglycerides; TRPI, troponin I; URBC, urinary red blood cell; UWBC, urinary white blood cell; WBC, white blood cell; X25HyD3, calcidiol.

Depression PGS were screened for associations with 315 clinical laboratory measurements using a LabWAS^38^ in VUMC’s biobank (N = 72 634). After multiple testing correction, the LabWAS of depression PGS revealed significant associations with 4 elevated immune markers: WBC (*P* = 1.07 × 10^−17^; β, 0.03; SE, 0.004), urinary WBC (*P* = 1.45 × 10^−5^; β, 0.03; SE, 0.007), absolute monocyte count (*P* = 2.54 × 10^−5^; β, 0.02; SE, 0.005), and absolute neutrophil count (*P* = 5.91 × 10^−5^; β, 0.02; SE, 0.005). Significant associations also included several metabolic markers, including increased triglycerides (*P* = 3.14 × 10^−18^; β, 0.05; SE, 0.006), decreased high-density lipoprotein cholesterol (*P* = 1.23 × 10^−11^; β, −0.04; SE, 0.005), decreased calcitriol (*P* = 2.83 × 10^−8^; β, –0.04; SE, 0.007), increased glucose (*P* = 2.84 × 10^−7^; β, 0.02; SE, 0.004), decreased blood urea nitrogen (*P* = 5.19 × 10^−7^; β, –0.02; SE, 0.004), decreased calcium (*P* = 9.74 × 10^−7^; β, –0.02; SE, 0.004), and decreased calcidiol (*P* = 7.03 × 10^−5^; β, –0.04; SE, 0.01). Depression PGS were also associated with decreased troponin I (*P* = 1.09 × 10^−6^; β, −0.05; SE, 0.009), decreased urinary red blood cells (*P* = 1.37 × 10^−5^; β, −0.03; SE, 0.006), decreased thyroxine (*P* = 1.72 × 10^−5^; β, −0.03; SE, 0.006), and decreased blood carbon dioxide (*P* = 4.06 × 10^−6^; β, −0.02; SE, 0.003) (Figure 1A; eTable 1 in Supplement 2).

In a conditional analysis, we sequentially controlled for diagnoses for depression, anxiety, adjustment reaction, and tobacco use disorder and for median body mass index across the EHR. In the analysis with all covariates, the most significant association remained WBC count (*P* = 1.11 × 10^−10^; β, 0.03; SE, 0.005), followed by triglycerides (*P* = 1.91 × 10^−5^; β, 0.04; SE, 0.008) (Figure 1B; eTables 2-6 in Supplements 3-7, respectively, and eFigures 1-2 in Supplement 1).

Although depression PGS remained robustly associated with WBC across all analyses, the magnitude of the association was modest (β, 0.03; SE, 0.004). Stratification of individuals in the discovery cohort (VUMC) showed that even at the highest decile of depression PGS, WBC measurements were elevated but remained within the clinical reference range (ie, 4-11 thousand cells/μL) (eFigure 3 in Supplement 1).

No laboratory results were significantly associated in the LabWAS of depression PGS in individuals of African descent, likely owing to the smaller sample size of the African ancestry sample (n = 12 383) and the low generalizability of PGS built using European summary statistics in African populations.^48^ However, the association with WBC count was in the same direction as in the European sample (*P* = .06; β, 0.02; SE, 0.01) (eFigure 4 in Supplement 1; eTable 8 in Supplement 9).

### Conditional Analyses of WBC

**Figure 2.**
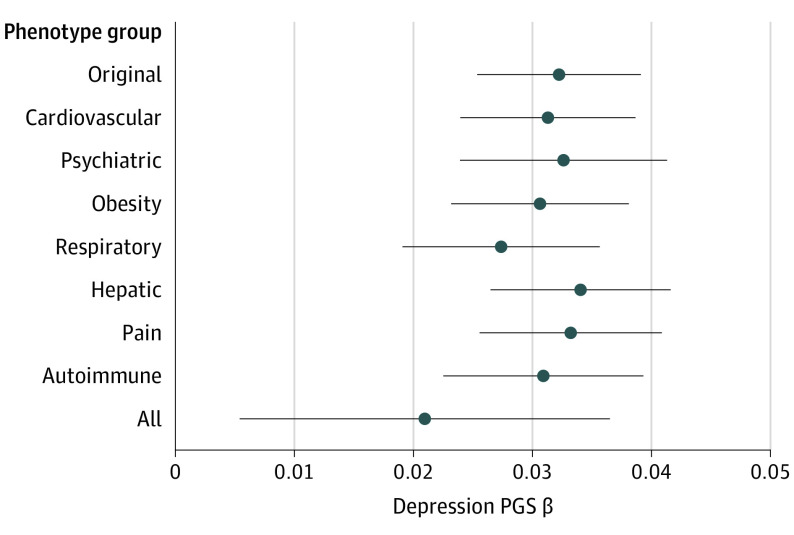
Analyses Controlled for Common Phenotypes Between Depression Polygenic Scores (PGS) and White Blood Cell (WBC) Count The association between depression PGS and WBC controlling for each “confounder” phenotype group in Vanderbilt University Medical Center. Group-based cases were any individual who was a case for any of a group’s phecodes and controls were individuals who were controls for all of a group’s phecodes. Associations were found using linear regressions controlled for each group. In the “All” analysis, all groups were controlled for in 1 regression. Whiskers indicate 95% CIs.

In separate phenome-wide association scans, depression PGS and median WBC count were significantly associated with 66 and 469 phecodes, respectively. Of these significantly associated phecodes, 32 were common to both depression PGS and median WBC count and were binned into 7 categories based on phenotypic similarity: cardiovascular, psychiatric, obesity, respiratory, hepatic, pain, and autoimmune conditions (Figure 2; eTable 9 in Supplement 1).

The association between depression PGS and WBC count remained significant after controlling for each group separately and controlling for all phenotype groups together (*P* = 4.19 × 10^−3^; β, 0.02; SE, 0.008) with effect estimates similar to the original association despite the reduced sample size (N = 13 269) (Figure 2; eTable 10 in Supplement 1).

### Replication in the PsycheMERGE Network

**Figure 3.**
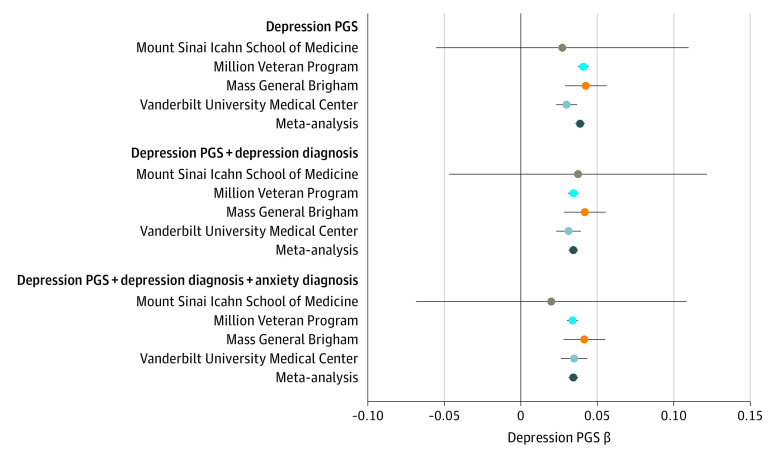
Replication Within the PsycheMERGE Network The association between depression polygenic scores (PGS) and median white blood cell levels was replicated across the PsycheMERGE Network with sensitivity analyses controlling for depression and anxiety diagnoses. Whiskers indicate 95% CIs.

Given the robustness of the association with WBC count and the history of associations between depression status and proinflammatory markers, we focused on WBC count for replication and further investigation. Findings were replicated in the 3 external biobanks (MVP, MSSM, and MGB) (Table 1). In both MVP (N = 289 880) and MGB (N = 20 828), the association between depression PGS and WBC remained significant with effect estimates replicating those observed at VUMC (Figure 3). In MSSM, the effect size point estimate was similar to those observed in the 3 other sites, but did not reach statistical significance, probably due to the smaller sample size (n = 823). The meta-analyzed effect estimate from the 4 sites was robust and significant (*P* = 1.03 × 10^−136^; β, 0.03; SE, 0.002), even after controlling for depression diagnosis (*P* = 9.52 × 10^−102^; β, 0.03; SE, 0.002) and after controlling for depression and anxiety diagnoses (*P* = 8.23 × 10^−100^; β, 0.03; SE, 0.002) (Figure 3; eTable 11 in Supplement 1).

**Table 1.** Characteristics of PsycheMERGE Network Sites

Site and group	No. of individuals genotyped (European)^a^	No. with WBC measurement	No. (%) genotyped and with WBC measurement	No. (%) female	No. (%) male	Mean age (SD), y	Mean (SD) length of record, y
Icahn School of Medicine at Mount Sinai							
All	9255	3668	823 (8.9)	429 (52.1)	394 (47.9)	59.7 (16.0)	11.2 (4.4)
Depression or anxiety controls	6722	2499	578 (8.6)	297 (51.4)	281 (48.6)	59.3 (16.4)	10.7 (4.4)
Depression or anxiety cases	1622	1169	245 (15.1)	132 (53.9)	113 (46.1)	60.5 (15.0)	12.5 (3.9)
Vanderbilt University Medical Center							
All	72 828	948 590	70 921 (97.4)	39 661 (55.9)	31 260 (44.1)	48.1 (22.3)	8.7 (6.3)
Depression or anxiety controls	59 520	301 982	43 129 (72.5)	22 675 (52.6)	20 454 (47.4)	46.8 (23.7)	7.6 (6.1)
Depression or anxiety cases	15 985	71 692	13 371 (83.6)	8614 (64.4)	4757 (35.6)	50.9 (18.8)	11.3 (6.1)
Million Veteran Program							
All	289 880	289 880	289 880 (100)	20 871 (7.2)	269 009 (92.8)	64.3 (12.0)	12.0
Depression or anxiety controls	150 328	150 328	150 328 (100)	6163 (4.1)	144 165 (95.9)	67.7 (11.2)	11.2
Depression or anxiety cases	129 552	129 552	129 552 (100)	14 121 (10.9)	115 431 (89.1)	61.6 (11.9)	12.9
Mass General Brigham							
All	25 331	72 329	20 828 (82.2)	10 726 (51.5)	10 102 (48.5)	56.1 (16.7)	13.8 (8.3)
Depression or anxiety controls	17 879	51 612	17 098 (95.6)	8891 (52.0)	8207 (48.0)	59.8 (16.7)	11.3 (7.1)
Depression or anxiety cases	7452	20 717	3730 (50.1)	2390 (64.2)	1340 (35.9)	56.7 (16.9)	14.0 (6.7)

Abbreviations: NA, not applicable; WBC, white blood cell.

^a^ The focused WBC analyses included only European samples, because there were no significant findings in the African ancestry LabWAS.

### Mediation Analysis

Two potential pathways between depression PGS, WBC count, and depression diagnosis were assessed using mediation analyses. In the first analysis, median WBC count was modeled as a mediator of the relationship between depression PGS (exposure) and depression diagnosis (outcome). Meta-analysis across all sites revealed that WBC count mediated 2.5% of the association between depression PGS and depression diagnosis (95% CI, 2.2%-20.8%; *P* = 2.84 × 10^−70^) (Table 2; eTable 12 in Supplement 1). When excluding MVP from the meta-analysis, WBC count mediated 0.5% of the association, although this was not a statistically significant association (95% CI, –0.03% to 0.9%; *P* = .06).

**Table 2.** White Blood Cell^a^ and Depression Diagnosis Mediation Analysis

Exposure	Mediator	Outcome	Site	*P* value	Proportion mediated (SE)	95% CI
Depression PGS	White blood cell count	Depression diagnosis	MGB	.01	0.012 (0.006)	(0.003 to 0.024)
			MVP	<2.23 × 10^−308^	0.035 (0.002)	(0.031 to 0.038)
			MSSM	.87	–0.016 (0.069)	(–0.242 to 0.118)
			VUMC	.14	0.003 (0.003)	(–0.001 to 0.008)
			Meta-analysis	2.84 × 10^−70^	0.025 (0.001)	(0.022 to 0.208)
Depression PGS	Depression diagnosis	White blood cell count	MGB	.01	0.044 (0.033)	(0.011 to 0.108)
			MVP	<2.23 × 10^−308^	0.162 (0.009)	(0.144 to 0.180)
			MSSM	.73	–0.104 (0.517)	(–1.511 to 0.910)
			VUMC	.15	0.01 (0.011)	(–0.004 to 0.032)
			Meta-analysis	1.78 × 10^−44^	0.098 (0.007)	(0.084 to 0.111)

Abbreviations: MGB, Mass General Brigham; MSSM, Icahn School of Medicine at Mount Sinai; MVP, Million Veteran Program; PGS, polygenic scores; VUMC, Vanderbilt University Medical Center.

^a^ White blood cell was investigated as a mediator between depression PGSs and depression diagnosis defined as phecode 296.2. Next, depression diagnosis was assessed as a mediator between depression PGSs and white blood cell. The proportion mediated was calculated by comparing the 50th percentile of depression PGSs to the 90th percentile.

In the second analysis, depression diagnosis was modeled as a mediator of the association between the depression PGS (exposure) and median WBC count (outcome). Meta-analysis across all sites indicated that depression diagnosis mediated 9.8% of the association between depression PGS and WBC count (95% CI, 8.4%-11.1%; *P* = 1.78 × 10^−44^) (Table 2; eTable 13 in Supplement 1). A depression diagnosis mediated 1.4% of the association when excluding MVP from the meta-analysis (95% CI, –0.6% to 3.4%; *P* = .17).

### Depression PGS and WBC-Differential Mediation Analysis

White blood cell counts are calculated from the sum of 5 different cell subtypes: neutrophils, lymphocytes, monocytes, basophils, and eosinophils. These cell subtypes can be measured along with the total WBC count using a complete blood count differential laboratory panel. To determine whether specific WBC components accounted for the associations between depression PGS and depression diagnosis, we performed a series of multiple mediator analyses.

When depression PGS was modeled as the exposure and depression diagnosis as the outcome, neutrophils were the only cell type that explained a significant proportion (1.9%; 95% CI, 0.2%-3.1%) of the association between depression PGS and depression diagnosis (eTable 14 in Supplement 1).

### Mendelian Randomization

When modeling WBC count as the exposure and depression as the outcome, mendelian randomization analysis provided additional evidence for an increase in depression risk with an increase in WBC (*P* = .01; estimated effect of the exposure on the outcome, 0.27) (eFigure 6; eTable 15 in Supplement 1). However, depression modeled as the exposure showed no evidence of a potential causal influence on the WBC outcome (*P* = .30; estimated effect of the exposure on the outcome, 0.022).

## Discussion

Depression is consistently associated with increased proinflammatory biomarkers; however, the mechanisms underlying these associations remain unclear. In this genetic association study, analysis of EHR-linked biobanks within the PsycheMERGE Network were used to examine the association between depression PGS and a variety of clinical laboratory traits, revealing a robustly replicated association with increased WBC count. Notably, several other laboratory traits were associated with depression PGS, including lipids, blood glucose, and blood urea nitrogen. The variety of associations with depression PGS suggest that multiple areas of biology are affected by depression genetics, including metabolism^49,50^ and inflammation.^50,51,52^ We chose to further investigate the association with WBC count given the existing literature and the robustness of the observed association with clinical confounders.

In a laboratory-wide screen, increased polygenic depression risk was associated with increased inflammatory markers, including WBC count, even after controlling for depression, anxiety, multiple comorbid phenotypes, body mass index, and smoking, thus suggesting that depression PGS was an important risk factor for the proinflammatory state observed in depression. These results suggested that genetic risk for depression, independent of depressive symptoms, was linked to a proinflammatory biomarker. The association of the depression PGS with WBC was modest across all biobanks, suggesting that individuals with high depression genetic liability may have an activated but not abnormal immune system. Nonetheless, sustained activation of the immune system could have important implications for the risk of developing depression.

There are 2 main models that connect depression to a proinflammatory state: the neuroinflammation model and the stress response model. The neuroinflammation model hypothesizes that an activated immune system contributes to risk of developing depression.^53,54^ The stress response model proposes the stress of depression symptoms leads to a proinflammatory state.^55,56^ Importantly, these 2 models are not mutually exclusive, and some have suggested they form a feedback loop.^57,58^ In support of this hypothesis, our mediation results do not distinguish either the neuroinflammation model or the stress response model as the exclusive pathway between depression and WBC count. However, mendelian randomization results supported a potential causal path from increased WBC levels to increased depression risk, consistent with the neuroinflammation model; but did not support a model of depression leading to increased WBC levels. It is important to note that only 47 SNVs met criteria to be included as depression instrument variables, limiting the statistical power of the analysis.

The notable difference in the proportions mediated between MVP and the other sites could be due to phenotypic uniqueness of the MVP sample. For example, the MVP comprises mostly male patients (92.8%), which could contribute to residual confounding by sex that is not fully accounted for in the model. Additionally, the mediated pathways could be particularly strong in MVP owing to the high prevalence of depression in the sample (MVP = 44.7%, others = 23.3%). A sensitivity analysis excluding MVP yielded marginally significant results, which indicates that an additional analysis in a larger sample size is warranted.

In the clinic, WBC measurements can be broken down into measurements of each WBC subtype. Abnormal levels of different WBC subtypes can index different immune processes. Understanding which cell types underlie the relationship between depression PGS and depression diagnosis through WBC can help narrow a specific immune process involved in depression. Neutrophil counts explained 1.9% of the association between depression PGS and depression diagnosis, and no other subtypes contributed to the association. Neutrophils are well known as responders to acute bacterial infection^59^ and are the most abundant WBC subtype in circulation (40%-60%).^59^ Recent evidence demonstrates that neutrophils have essential roles in innate and adaptive immunity,^60^ are implicated in diseases of chronic inflammation,^61^ and are experimentally shown to transmigrate into intact mouse brain to deliver interleukin 1β, resulting in depressive behavioral change.^62^

### Limitations

Our study should be interpreted in light of its limitations. First, the WBC measurements used in the study were clinically derived, with measurements reflecting a range of health states. To address this limitation, we limited to observations within 4 SDs (eMethods in Supplement 1) and noted that WBC count was measured for nearly everyone in our primary and replication sample populations. However, it remains possible that individuals with clinical orders for WBC-differential panels may represent a clinically different sample than those with only the total WBC measurement. Additionally, EHRs often contain multiple WBC measurements for the same individual. In this study, only the median values per individual were used, leaving unanswered questions about the effect of depression PGS on WBC count over time and in response to antidepressant treatment. Second, the depression PGS are based on genome-wide association statistics that are not adjusted for phenotypic comorbidities such as proinflammatory conditions. This situation introduces the possibility of “phenotypic hitchhiking” in which a comorbid trait is unintentionally selected during the ascertainment of the index trait. This unintentional selection would create a situation in which 2 heritable phenotypes that share common environmental risk factors but no genetic risk factors can appear correlated in PGS analysis, even in independent samples. Therefore, we emphasize that the PGS approach is still fundamentally an association. Third, results are based on genetic studies of primarily European ancestry populations and may not generalize across diverse ancestries. Fourth, while pleiotropy was assessed in the mendelian randomization analyses, possible unknown sources of confounding (such as those mentioned in the description of “phenotypic hitchhiking”) were not assessed. Finally, even though the association between depression PGS and WBC count was robust, the effect sizes were small, making WBC count an unlikely candidate for use as a diagnostic biomarker of depression.

## Conclusions

In this genetic association study, PGS for depression were associated with increased inflammatory markers, specifically WBC count, even in the absence of depressive symptoms. The associations described in this study highlight the importance of WBC biology in depression and demonstrate the potential use of EHR-based genomics as a tool for discovery of physiological markers in psychiatric traits.

## Notes

Supplement 1.
eMethods

eResults
**eFigure 1.** LabWAS of Depression PGS in VUMC **eFigure 2.** Volcano Plots of Depression PGS LabWAS in VUMC **eFigure 3.** Median WBC Measurements Stratified by Depression PGS Decile in VUMC **eFigure 4.** Lab-Wide Association Scan of Depression PGS in Individuals of African Ancestry in VUMC **eFigure 5.** Controlling for the Impact of WBC Genetics on the Association Between Depression PGS and WBC Levels **eFigure 6.** Results of Bidirectional Mendelian Randomization **eTable 9.** Phenotypes Associated With Depression PGS and Median WBC Measurements Using PheWAS in VUMC **eTable 10.** Association Between Depression PGS and WBC Levels Controlled for Common Phenotype Groups in VUMC **eTable 11.** Results of PsycheMERGE Replication Between Depression PGS and WBC Levels **eTable 12.** Mediation Results With WBC as the Mediator Across PsycheMERGE Sites **eTable 13.** Mediation Results With MDD Diagnosis as the Mediator Across PsycheMERGE Sites **eTable 14.** Immune Subpopulation and Depression Diagnosis Mediation Analysis **eTable 15.** Results of Bidirectional Mendelian Randomization **eTable 16.** Results of Controlling for WBC Genetics Across PsycheMERGE Sites
eReferences.

Supplement 2. **eTable 1.** LabWAS of Depression PGS in European Sample in VUMC

Supplement 3. **eTable 2.** LabWAS of Depression PGS Controlled for Depression Diagnosis in European Sample in VUMC

Supplement 4. **eTable 3.** LabWAS of Depression PGS Controlled for Depression and Anxiety Diagnoses in European Sample in VUMC

Supplement 5. **eTable 4.** LabWAS of Depression PGS Controlled for Depression, Anxiety, and Adjustment Reaction Diagnoses in European Sample in VUMC

Supplement 6. **eTable 5.** LabWAS of Depression PGS Controlled for Median BMI, Depression, Anxiety, and Adjustment Reaction Diagnoses in European Sample in VUMC

Supplement 7. **eTable 6.** LabWAS of Depression PGS Controlled for Median BMI, Depression, Anxiety, Adjustment Reaction, and Tobacco use Disorder Diagnoses

Supplement 8. **eTable 7.** LabWAS of Depression PGS Controlled for Median BMI, Depression, Anxiety, Adjustment Reaction Diagnoses, and Ever Smoked in European Sample in VUMC

Supplement 9. **eTable 8.** LabWAS of Depression PGS in African Sample in VUMC
