# Supplementary material for: Use of the PsycheMERGE Network to Investigate the Association Between Depression Polygenic Scores and White Blood Cell Count
Source: JAMA Psychiatry. 2021 Oct 20;78(12):1–10. doi: 10.1001/jamapsychiatry.2021.2959 (PMC8529528; doi:10.1001/jamapsychiatry.2021.2959)
Supplement: Supplement 1. — eMethods eResults eFigure 1. LabWAS of Depression PGS in VUMC eFigure 2. Volcano Plots of Depression PGS LabWAS in VUMC eFigure 3. Median WBC Measurements Stratified by Depression PGS Decile in VUMC eFigure 4. Lab-Wide Association Scan of Depression PGS in Individuals of African Ancestry in VUMC eFigure 5. Controlling for the Impact of WBC Genetics on the Association Between Depression PGS and WBC Levels eFigure 6. Results of Bidirectional Mendelian Randomization eTable 9. Phenotypes Associated With Depression PGS and Median WBC Measurements Using PheWAS in VUMC eTable 10. Association Between Depression PGS and WBC Levels Controlled for Common Phenotype Groups in VUMC eTable 11. Results of PsycheMERGE Replication Between Depression PGS and WBC Levels eTable 12. Mediation Results With WBC as the Mediator Across PsycheMERGE Sites eTable 13. Mediation Results With MDD Diagnosis as the Mediator Across PsycheMERGE Sites eTable 14. Immune Subpopulation and Depression Diagnosis Mediation Analysis eTable 15. Results of Bidirectional Mendelian Randomization eTable 16. Results of Controlling for WBC Genetics Across PsycheMERGE Sites eReferences. [file jamapsychiatry-e212959-s001.pdf]

Supplementary Online Content

Sealock JM, Lee YH, Moscati A, et al. Use of PsycheMERGE network to investigate the association between depression polygenic scores and white blood cell count. *JAMA Psychiatry*. Published online October 20, 2021. doi:10.1001/jamapsychiatry.2021.2959

eMethods.
eResults.
eFigure 1. LabWAS of Depression PGS in VUMC
eFigure 2. Volcano Plots of Depression PGS LabWAS in VUMC
eFigure 3. Median WBC Measurements Stratified by Depression PGS Decile in VUMC
eFigure 4. Lab-Wide Association Scan of Depression PGS in Individuals of African Ancestry in VUMC
eFigure 5. Controlling for the Impact of WBC Genetics on the Association Between Depression PGS and WBC Levels
eFigure 6. Results of Bidirectional Mendelian Randomization
eTable 9. Phenotypes Associated With Depression PGS and Median WBC Measurements Using PheWAS in VUMC
eTable 10. Association Between Depression PGS and WBC Levels Controlled for Common Phenotype Groups in VUMC
eTable 11. Results of PsycheMERGE Replication Between Depression PGS and WBC Levels
eTable 12. Mediation Results With WBC as the Mediator Across PsycheMERGE Sites
eTable 13. Mediation Results With MDD Diagnosis as the Mediator Across PsycheMERGE Sites
eTable 14. Immune Subpopulation and Depression Diagnosis Mediation Analysis
eTable 15. Results of Bidirectional Mendelian Randomization
eTable 16. Results of Controlling for WBC Genetics Across PsycheMERGE Sites
eReferences.

This supplementary material has been provided by the authors to give readers additional information about their work.

© Sealock JM et al. *JAMA Psychiatry.*

eMethods

PsycheMERGE Study Samples

Vanderbilt University Medical Center

Vanderbilt University Medical Center (VUMC) is a tertiary care center that provides inpatient and outpatient care in Nashville, TN. The VUMC electronic health record (EHR) system was established in 1990 and includes data on billing codes from the International Classification of Diseases, 9th and 10th editions (ICD-9 and ICD-10), Current Procedural Terminology (CPT) codes, laboratory values, reports, and clinical documentation. In 2007, VUMC launched a biobank, BioVU, which links a patient's DNA sample to their EHR. The BioVU consent form is provided to patients in the outpatient clinic environments at VUMC. The form states policies on data sharing and privacy, and should a signature be obtained, makes any blood leftover from clinical care eligible for BioVU banking. The VUMC Institutional Review Board oversees BioVU and approved this project (IRB#172020) In VUMC, primary analyses were conducted in individuals of European ancestry, with a sensitivity analysis in individuals of African ancestry.

Million Veteran Program

The Million Veteran Program is an observational cohort study and mega-biobank in the Department of Veterans Affairs (VA)[1]. Participants are active users of the Veterans Health Administration and provide a blood sample, responses to questionnaires and consent to allow access to clinical data from the VA electronic health records[1]. The MVP v3.0 data release used in this study includes genotyping data from 455,789 individuals; DNA was extracted from whole blood (which was collected during enrollment to the MVP) and genotyping was performed with the MVP 1.0 Genotyping array[1]. For this study, we only considered samples with a European Ancestry (EUR) as determined by HARE (Harmonized ancestry and race/ethnicity) analysis[2].

Icahn School of Medicine at Mount Sinai

The Bio*Me* Biobank, at the Icahn School of Medicine at Mount Sinai, is an EHR-linked biobank of participants from the Mount Sinai Health System in New York, NY. Participant recruitment into Bio*Me* has been ongoing since 2007, predominantly recruited from general medicine and primary care clinics, and the rest from specialty practices and recruitment events.
Bio*Me* participants consent to provide DNA and plasma samples linked to their de-identified EHRs, and then provide additional information on self-reported ancestry, health behaviors, and medical history through questionnaires administered upon enrollment.

Massachusetts General Brigham Biobank (MGBB)

The Massachusetts General Brigham Biobank, formerly known as the Partners Healthcare Biobank, is an ongoing virtual cohort study of patients across the MGB General Brigham hospital system (including Brigham and Women's Hospital, Massachusetts General Hospital, and other affiliated hospitals), which provides a large-scale resource of linked longitudinal electronic health records (EHR) data, genomic data, and self-reported survey data[3]. All patients provided informed consent before enrollment, and all study procedures were approved by the Massachusetts General Brigham Institutional Review Board.

Lab Quality Control

Labs were required to have at least 70% of observations in a single set of units and filtered for at least 1,000 observations over at least 100 individuals. Observations outside 4 standard deviations of the sample mean were excluded to remove extreme values including those that are biologically implausible. The median observation for each individual in each lab was selected and adjusted for cubic splines of age at measurement. The age-adjusted value was normalized using a rank-based inverse normal transformation (INT) to ensure a normal distribution for downstream analyses, generating age-adjusted INT lab values. For genetic analysis, labs exhibiting no measurable heritability through the GREML analysis in the GCTA software[4] were excluded, leaving 315 labs for use.

Phecode Defintions

Phecodes are used in phenome-wide association scans (PheWAS). In a PheWAS, ICD codes are hierarchically grouped together into 'phecodes' based on phenotypic similarity. In LabWAS sensitivity analyses, we controlled for depression diagnosis, anxiety diagnosis, and adjustment reaction disorder, defined as phecode 296.2, 300.1, and 304, respectively. For all analyses involving phecodes, cases were required to have at least two instances of component ICD codes and controls were required to have zero component ICD codes and zero phecode exclusion codes defined by the phecode map in the PheWAS R package v0.99[5]. Individuals with only one component ICD code were excluded.

In PheWAS analyses conducted during sensitivity analyses in VUMC, phecodes were required to have at least 100 cases to be included in the scans.

Depression PGS and WBC Mediation Analysis

While mediation analysis can be easily performed with continuous exposures (in this case the MDD-PGS), the calculation of the "proportion of variance mediated" cannot be interpreted on a continuous scale. Instead, we have to specify two discrete levels of the exposure in order to make the contrast (i.e., average MDD-PGS and high MDD-PGS). Therefore, the reference level (average MDD-PGS) and the comparison level (high MDD-PGS) must be defined by two distinct levels of the exposure variable. We selected individuals in the 50th percentile to represent the average MDD-PGS and tested three different comparison levels including individuals at the 85th, 90th, and 95th percentiles. There was no meaningful difference in the proportion mediated between the three comparison levels, thus we chose the 90th percentile as representative of the "high MDD-PGS" in the main table and have provided all results in the supplementary table.

© Sealock JM et al. *JAMA Psychiatry.*

Depression PGS and WBC-differential Mediation Analysis

In a multiple mediator analysis, a single main mediator and additional alternative mediators are specified. A structural equation modeling approach is used to assess the effect of the main mediator between the exposure and outcome after controlling for the correlation structure between the alternative mediators and the outcome[6]. All measurements were required to be recorded on the same date for each individual to ensure they were from the same WBC-differential (N=24,383). For individuals with multiple WBC-differentials recorded in their EHR, median WBC values and the corresponding subtype absolute values were selected. All measurements were adjusted for cubic splines of age at observation and normalized[8,9].

Controlling for the impact of WBC genetics

We first tested the correlation between WBC PGS and depression diagnosis. The WBC PGS were constructed using PRS-CS-auto with the 1000 Genomes Project Phase 3 European subset reference panel and weights from the UK Biobank WBC summary statistics[10]. WBC PGS were z-score scaled so that the effect estimate is per standard deviation increase in PGS. We first confirmed that the WBC PGS was significantly associated with measured WBC ($p < 2.23 \times 10^{-308}$; beta = 0.14). Then we tested the association between WBC PGS and depression diagnosis (defined as phecode 296.2) across all biobanks, controlling for sex, median age across the medical record, and top 10 genetic principal components using a linear regression model.

In a separate series of analyses, the influence of WBC genetic factors within the depression PGS was examined. First, the effect of genetic regulation of WBC was adjusted from the depression summary statistics by conditioning the depression summary statistics on the WBC summary statistics using multi-trait-based conditional & joint analysis[11] (mtCOJO) in GCTA version1.91.4. Next, conditioned depression PGS were constructed using the conditioned depression summary statistics. Finally, we tested the association between the conditioned depression PGS and the median age-adjusted INT normalized WBC measurements, controlling for sex and top 10 principal components of ancestry. The effect estimates of each analysis from all four sites were meta-analyzed using a fixed-effect inverse variance weighted model in the meta[7] R package. Results are presented in the Supplementary Results, Supplementary Figure 5, and Supplementary Table 16.

eResults

Controlling for the impact of WBC genetics

The LabWAS and phe-group conditional analyses confirmed a correlation between increased depression PGS and elevated WBC that is unlikely to be due to an underlying comorbid confounder. However, there remain multiple possible hypotheses to explain these results. First, increased depression PGS may lead to elevated WBC through mechanisms that directly (e.g., hematopoiesis) or indirectly (e.g., cortisol) activate the immune system. However, given that WBC itself is a highly heritable trait (h^2 = 14 – 40%[12]), it is also possible that the GWAS used to train the depression PGS includes WBC genetic associations due to either phenotypic hitchhiking in the original ascertainment of depression, or a real contribution from the heritable component of WBC acting as an independent risk factor for depression. In either circumstance, this would result in depression cases having higher WBC PGS than controls, which would also be reflected in the depression PGS and the subsequent genetic correlation between WBC and depression in independent samples.

To address these complicating issues, we undertook a series of analyses. First, we calculated the genetic correlation between depression and WBC, which was non-significant (rg = 0.027, p-value = 0.268). Next, the weights for the depression PGS were conditioned on the weights from the WBC GWAS using mtCOJO to adjust for the effects of WBC on depression genetic risk. A conditional depression PGS built from the conditioned weights (MDD|WBC PGS) remained associated with WBC (p-value=3.60×10^{-108}, beta=0.035), confirming the majority of the association between depression PGS and measured WBC arises not from the heritable component of WBC, but from the impact of genes that increase risk for depression (Supplementary Figure 4, Supplementary Table 9).

Next, we tested the association between WBC PGS and depression diagnosis in each biobank. The meta-analysis across the four sites suggested a small though significant association between WBC PGS and depression (p-value = 3.52×10^{-5}, beta=0.015), but this association was only observed in the MVP which contributed the largest sample size to the meta-analysis. In VUMC, depression status was not significantly associated with WBC levels (p-value = 0.053, beta = 0.009, SE=0.01).

© Sealock JM et al. *JAMA Psychiatry.*

eFigure 1. LabWAS of Depression PGS in VUMC controlled for a) sex and top 10 principal components of ancestry, b) depression diagnosis, c) depression and anxiety diagnoses, d) depression, anxiety, and adjustment reaction, e) diagnoses for depression, anxiety, adjustment reaction, and median BMI, f) diagnoses for depression, anxiety, adjustment reaction, tobacco use disorder and median BMI, and g) diagnoses for depression, anxiety, adjustment reaction, median BMI, and smoking ever documented in the EHR. Depression, anxiety, adjustment reaction, and tobacco use disorder diagnoses were defined as phecodes 296.2, 300.1, 304, and 318, respectively. The red line indicates the Bonferroni threshold for statistical significance (p<1.58 x 10-4) and the blue line indicates a p-value of 0.05. Upward triangles indicate that the PGS is associated with increased levels of the lab, while

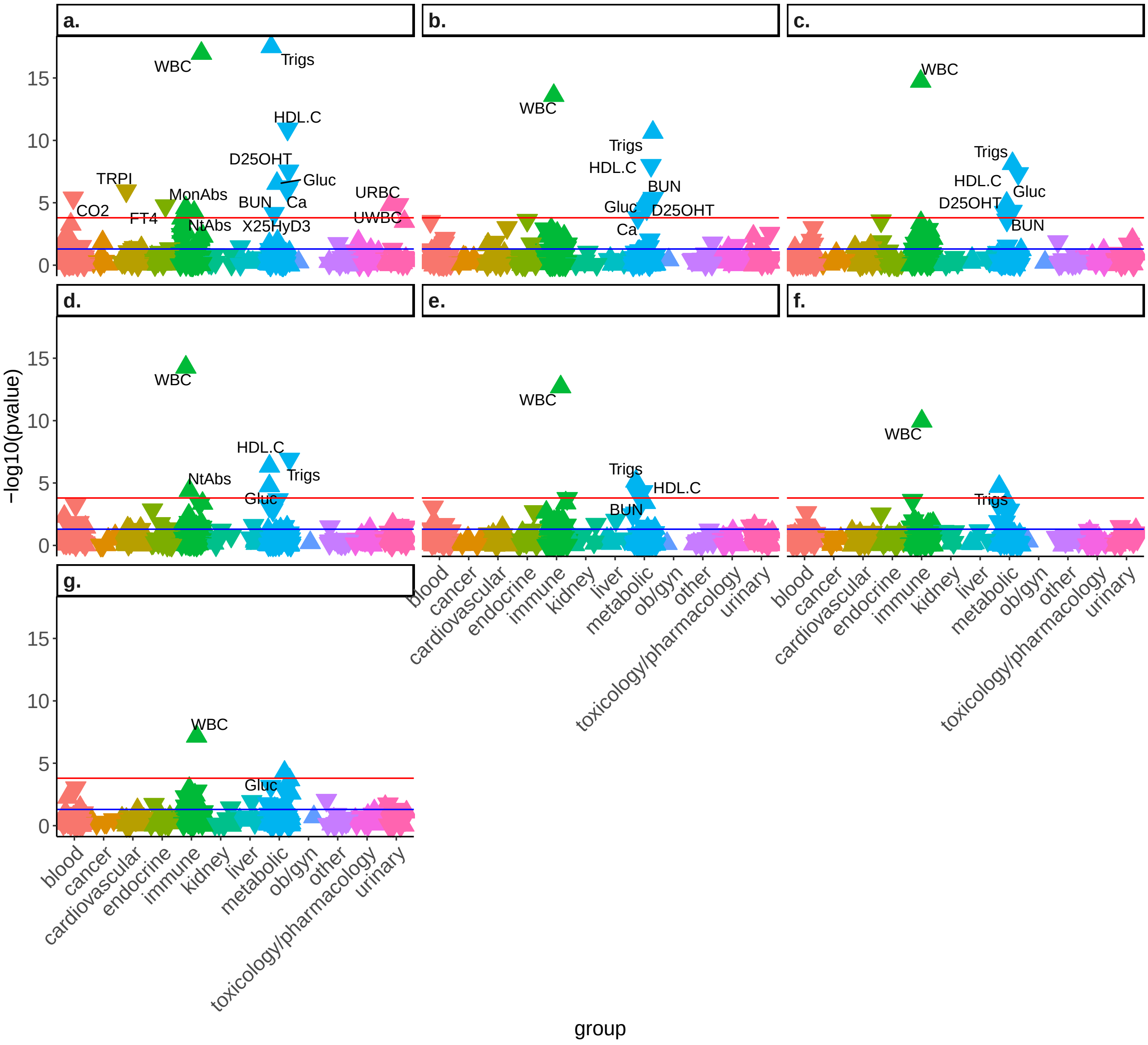

downward triangles indicate an association with reduced levels of the lab.

© Sealock JM et al. *JAMA Psychiatry.*

eFigure 2. Volcano Plots of Depression PGS LabWAS in VUMC controlled for a) sex and top 10 principal components of ancestry, b) depression diagnosis, c) depression and anxiety diagnoses, and d) diagnoses for depression, anxiety, and adjustment disorder and median BMI. Associations passing Bonferroni correction are denoted in red.

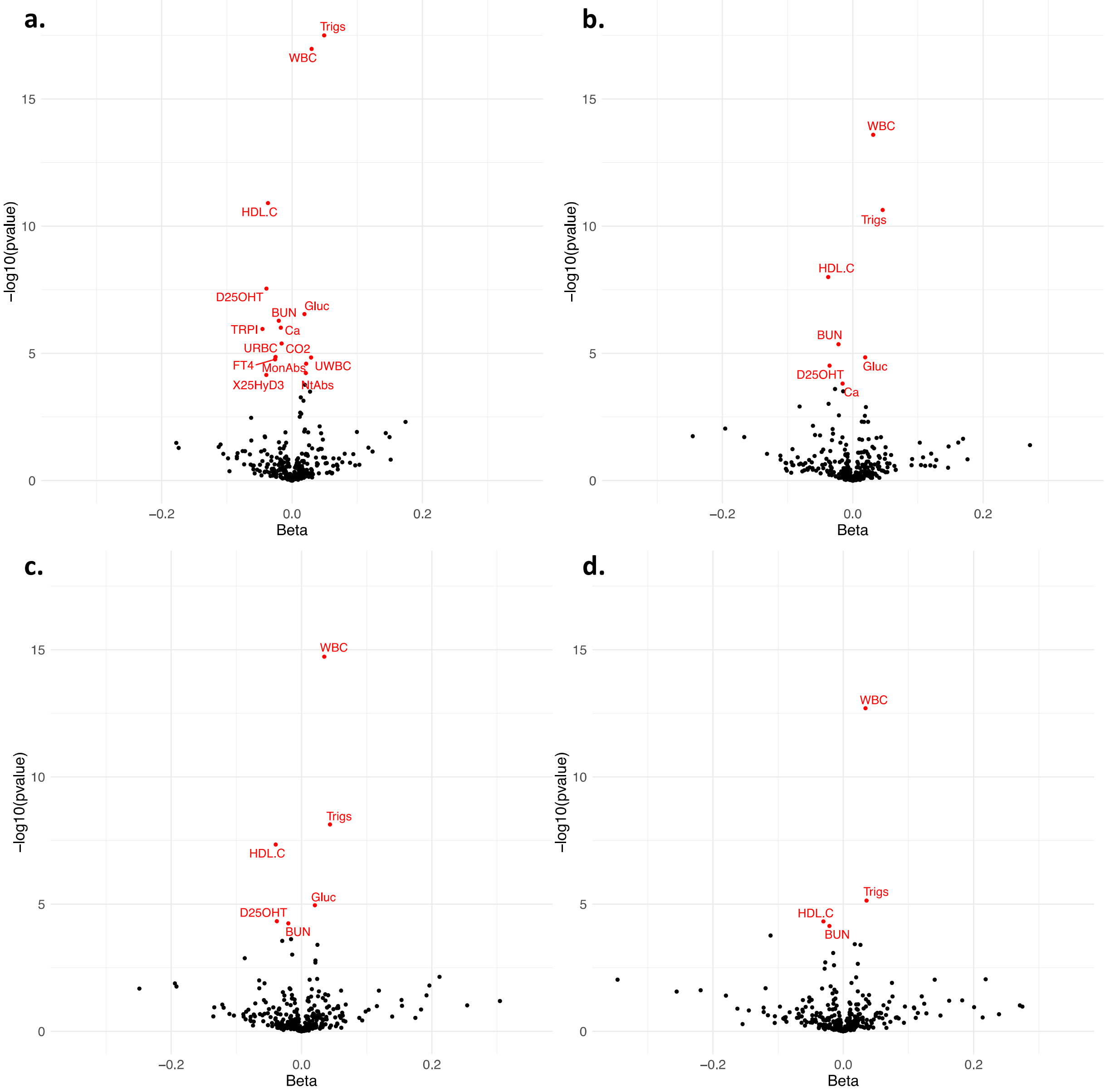

© Sealock JM et al. *JAMA Psychiatry.*

eFigure 3. Median WBC Measurements Stratified by Depression PGS Decile in VUMC
Individuals were divided into deciles based on their depression PGS. Each individual's median untransformed WBC measurement is plotted based on depression PGS decile. Blue lines indicate the normal clinical range for WBC (4-11thou cells/uL). The dotted line in between boxes connects median WBC values between deciles. b) Regression plot between median age-adjusted, inverse normalized WBC measurements and the residual of MDD PGS on sex and top 10 prinicpal components. The blue line represents the regression line between MDD PGS and WBC.

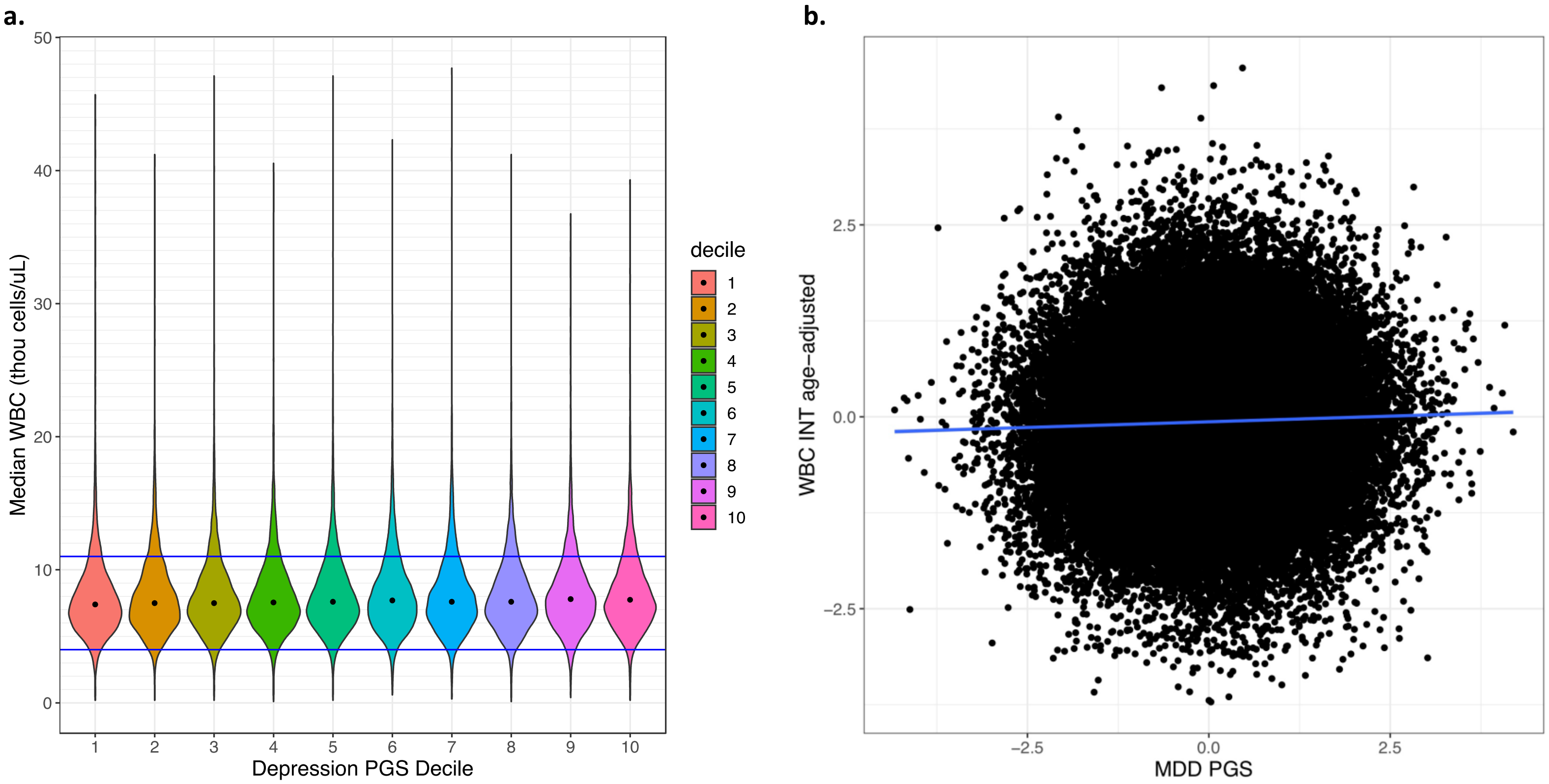

© Sealock JM et al. *JAMA Psychiatry.*

eFigure 4. Lab-Wide Association Scan of Depression PGS in Individuals of African Ancestry in VUMC

Associations were controlled for sex and top 1o principal components of ancestry. The blue line represents p-value = 0.05, and the red line represents Bonferroni signiciance (p-value=2.21 x 10^{-4}).

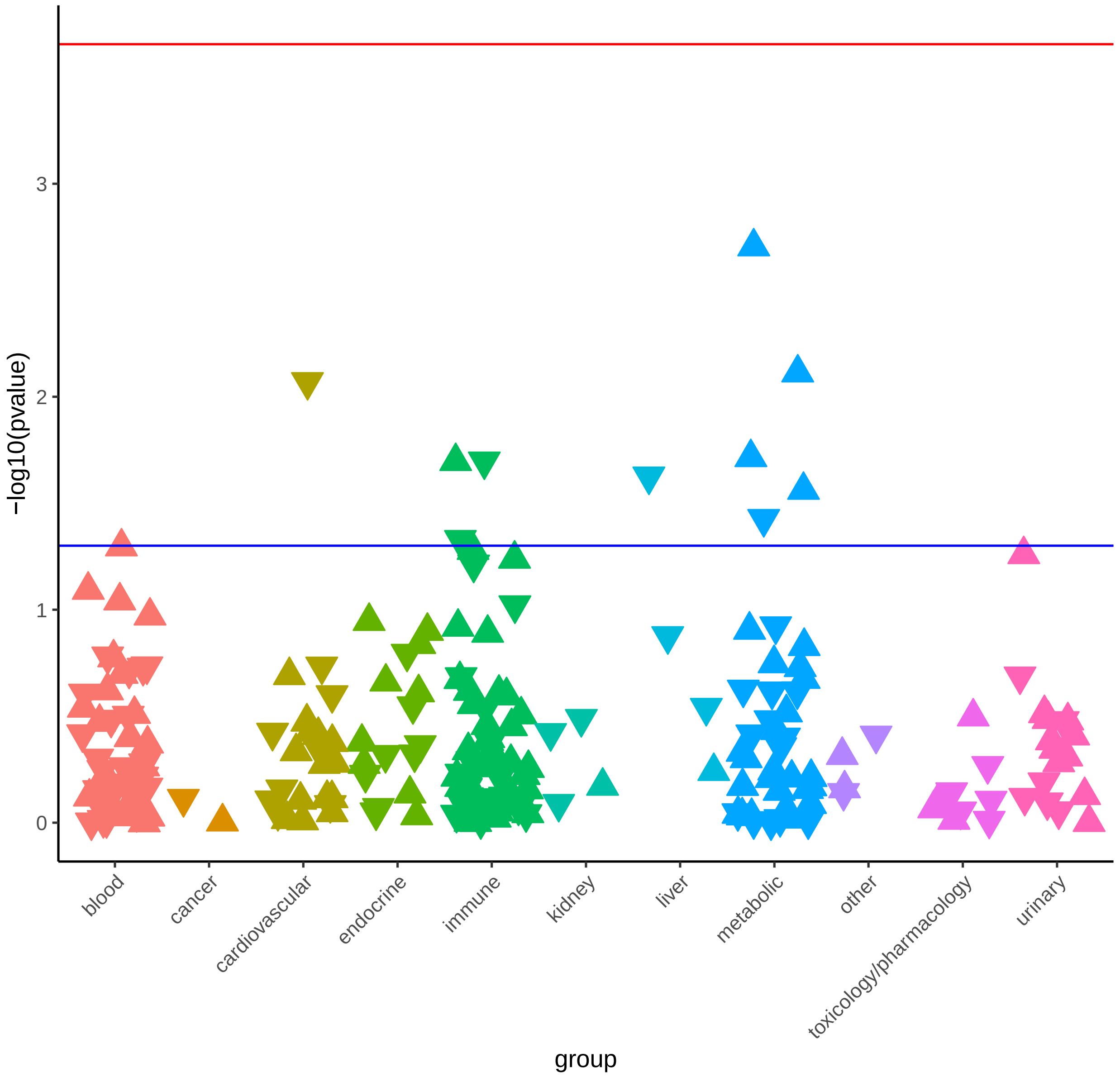

© Sealock JM et al. *JAMA Psychiatry.*

eFigure 5. Controlling for the Impact of WBC Genetics on the Association Between Depression PGS and WBC Levels

The pathway between WBC genetics and depression diagnosis was assessed by regressing a WBC polygenic score on depression diagnosis defined as phecode 296.2. The effect of WBC genetics in depression genetics was assessed by conditioning depression PGS on WBC genetics and finding the association with WBC measurements.

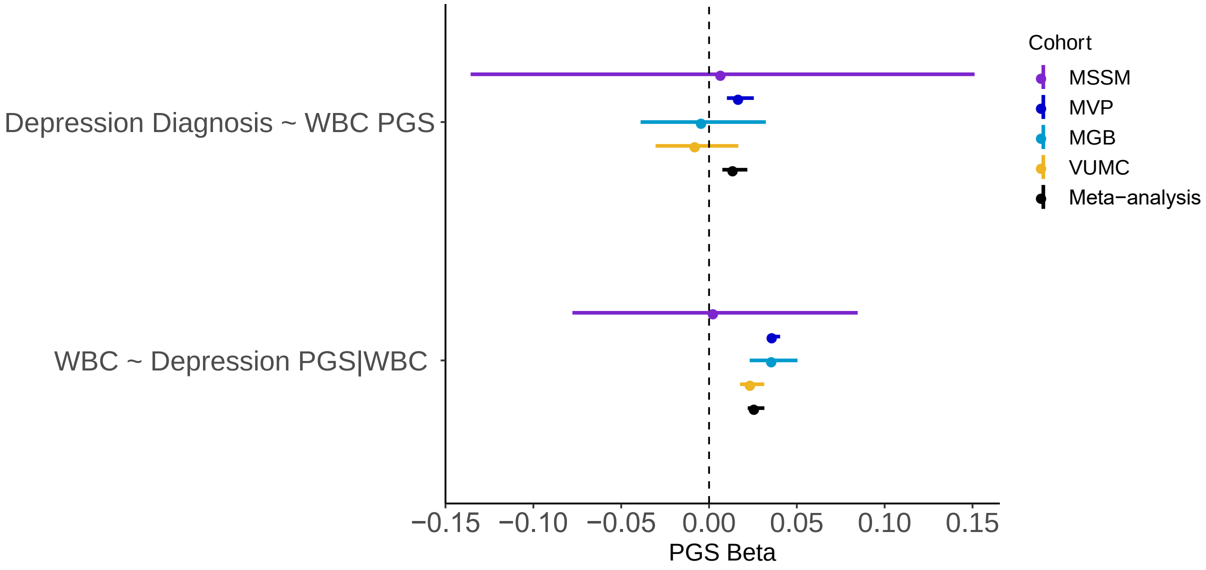

© Sealock JM et al. *JAMA Psychiatry.*

eFigure 6. Results of Bidirectional Mendelian Randomization with a) MDD as the exposure and WBC as the outcome, and b) WBC as the exposure and MDD as the outcome.

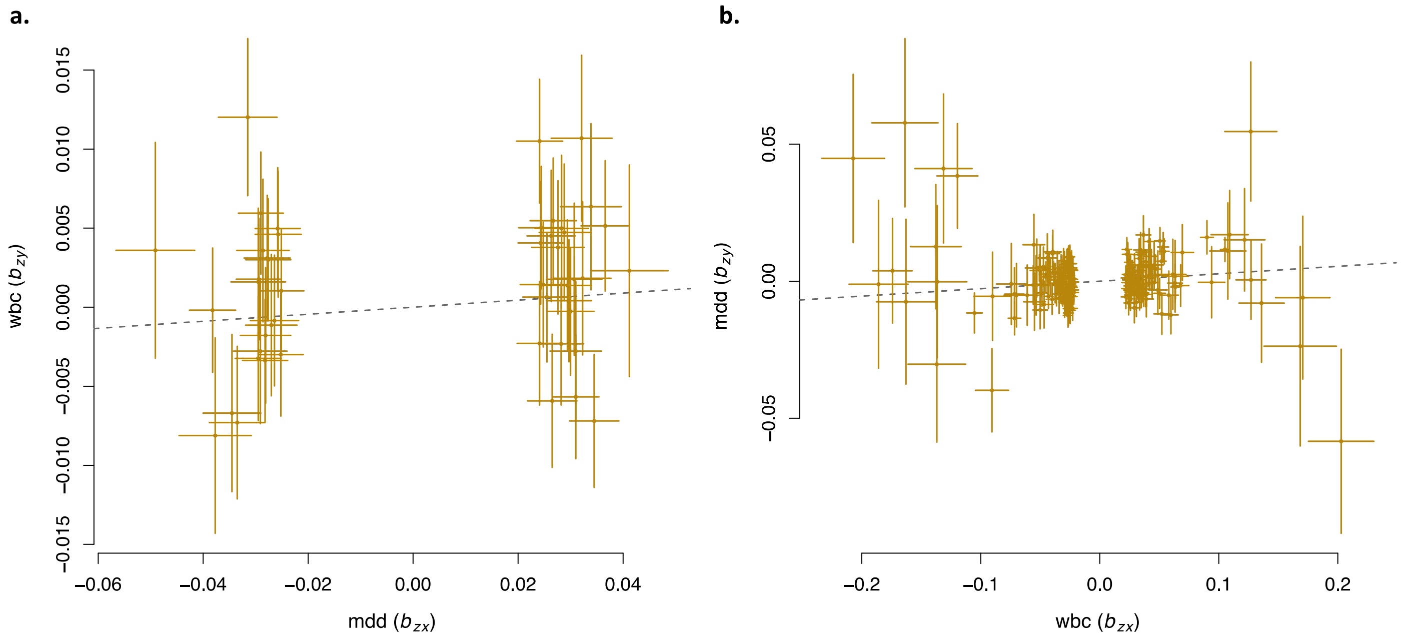

© Sealock JM et al. *JAMA Psychiatry.*

eTable 9. Phenotypes Associated With Depression PGS and Median WBC Measurements Using PheWAS in VUMC

Common phenotypes passing Bonferroni significance in each scan were binned into categories based on similarity.

Phecode	Phenotype	Groups	WBC p-value	WBC beta	Depression PGS p-value	Depression PGS beta
411.3	Angina pectoris	Cardiovascular	5.14E-20	0.0265	1.54E-06	0.0244
433	Cerebrovascular disease	Cardiovascular	1.09E-29	0.0176	6.37E-06	0.0157
428.1	Congestive heart failure (CHF) NOS	Cardiovascular	3.90E-69	0.017	2.38E-05	0.0152
411.4	Coronary atherosclerosis	Cardiovascular	2.34E-98	0.0151	1.12E-08	0.0131
401.1	Essential hypertension	Cardiovascular	3.26E-143	0.0115	4.90E-06	0.0099
401	Hypertension	Cardiovascular	6.38E-142	0.0114	9.73E-07	0.0098
411	Ischemic Heart Disease	Cardiovascular	5.21E-104	0.0144	7.38E-09	0.0125
411.2	Myocardial infarction	Cardiovascular	1.17E-78	0.0213	1.22E-05	0.0192
418	Nonspecific chest pain	Cardiovascular	5.14E-18	0.0116	1.97E-14	0.0105
411.1	Unstable angina (intermediate coronary syndrome)	Cardiovascular	9.82E-21	0.0274	1.95E-08	0.0252
292.4	Altered mental status	Psychiatric	1.29E-14	0.0194	1.91E-05	0.0176
313.1	Attention deficit hyperactivity disorder	Psychiatric	1.22E-12	0.0316	2.27E-07	0.0324
296.1	Bipolar	Psychiatric	9.90E-06	0.0263	3.91E-23	0.0261
313	Pervasive developmental disorders	Psychiatric	2.63E-15	0.0265	2.40E-08	0.0274
300.9	Posttraumatic stress disorder	Psychiatric	1.96E-06	0.0324	2.76E-25	0.0327
318	Tobacco use disorder	Psychiatric	4.77E-175	0.0152	1.89E-25	0.0137
250	Diabetes mellitus	Obesity	3.02E-100	0.013	1.62E-06	0.0116
278.11	Morbid obesity	Obesity	5.06E-90	0.021	5.71E-09	0.0197
278.1	Obesity	Obesity	4.41E-94	0.0151	9.65E-09	0.0139
278	Overweight, obesity and other hyperalimentation	Obesity	1.11E-81	0.0141	9.54E-08	0.013
250.2	Type 2 diabetes	Obesity	7.15E-110	0.0134	7.60E-08	0.0119
327.3	Sleep apnea	Respiratory	1.36E-14	0.0161	9.64E-06	0.0149
495	Asthma	Respiratory	9.18E-25	0.0181	3.86E-06	0.0171
496	Chronic airway obstruction	Respiratory	5.69E-144	0.018	1.68E-08	0.016
496.2	Chronic bronchitis	Respiratory	6.60E-55	0.0325	1.02E-05	0.03
512.7	Shortness of breath	Respiratory	1.59E-91	0.0132	6.99E-07	0.0117
70	Viral hepatitis	Hepatic	7.14E-53	0.0301	4.40E-07	0.0279
70.3	Viral hepatitis C	Hepatic	6.85E-52	0.0319	1.35E-06	0.0296
338.1	Acute pain	Pain	8.70E-23	0.0151	2.38E-06	0.0138
338	Pain	Pain	1.20E-20	0.0133	4.09E-09	0.0121

798	Malaise and fatigue	Pain	1.45E-63	0.0108	3.49E-07	0.0098
557.1	Celiac disease	Autoimmune	1.52E-15	0.0568	1.78E-09	0.0545

eTable 10. Association Between Depression PGS and WBC Levels Controlled for Common Phenotype Groups in VUMC

The association was controlled for each phenotype group separately and all groups in one analysis in the "All" phenotype. Associations were found using a linear regression controlling for sex and top 10 principal components of ancestry.

**Phenotype**	**N**	**Depression PGS P-value**	**Depression PGS beta**	**SE**	**Lower 95% CI**	**Upper 95% CI**
Original	65,120	1.07E-17	0.030	0.003	0.023	0.037
Cardiovascular	55,184	8.1E-17	0.031	0.004	0.024	0.039
Psychiatric	41,213	2.1E-13	0.033	0.004	0.024	0.041
Obesity	54,28	8.4E-16	0.031	0.004	0.023	0.038
Respiratory	44,274	9.7E-11	0.027	0.004	0.019	0.036
Hepatic	54,016	1.2E-18	0.034	0.004	0.027	0.042
Pain	52,649	1.9E-17	0.033	0.004	0.026	0.041
Autoimmune	44,504	5.5E-12	0.029	0.004	0.021	0.037
All	13,269	0.0082	0.021	0.008	0.005	0.037

eTable 11. Results of PsycheMERGE Replication Between Depression PGS and WBC Levels were controlled for sex and top 10 principal components. Beta estimates were combined for meta-analysis using a fixed-effects inverse weighted method.

Analysis	Cohort	P-value	Beta	SE	Lower 95% CI	Upper 95% CI
Depression PGS	MSSM	0.519	0.027	0.042	-0.055	0.11
	MVP	3.84E-114	0.041	0.002	0.037	0.044
	MGB	9.11E-10	0.043	0.007	0.029	0.056
	VUMC	1.07E-17	0.03	0.004	0.023	0.037
	Meta-analysis	1.03E-136	0.034	0.002	0.031	0.038
Depression PGS + Depression Diagnosis	MSSM	0.384	0.038	0.043	-0.047	0.122
	MVP	2.12E-81	0.035	0.002	0.031	0.038
	MGB	1.92E-9	0.042	0.007	0.028	0.056
	VUMC	2.54E-14	0.031	0.004	0.023	0.039
	Meta-analysis	9.52E-102	0.034	0.002	0.031	0.038
Depression PGS + Depression Diagnosis + Anxiety Diagnosis	MSSM	0.658	0.02	0.045	-0.069	0.109
	MVP	1.71E-78	0.034	0.002	0.03	0.037
	MGB	2.55E-9	0.042	0.007	0.028	0.055
	VUMC	1.88E-15	0.035	0.004	0.026	0.044
	Meta-analysis	8.23E-100	0.034	0.002	0.031	0.038

eTable 12. Mediation Results With WBC as the Mediator Across PsycheMERGE Sites mediated estimates were estimated using three different treatment percentiles of depression PGS (85%, 90%, and 95%) compared to the 50% percentile of PGS for controls.

**Cohort**	**MDD PGS Percentile**	**Control Percetile Value**	**Treatment Percentile Value**	**Proportion Mediated pvalue**	**Proportion Mediated (SE)**	**Lower 95% CI**
VUMC	0.85	0.011	1.033	0.138	0.003 (0.003)	-0.001 – 0.008
	0.90		1.279	0.138	0.003 (0.003)	-0.001 – 0.008
	0.95		1.634	0.138	0.003 (0.003)	-0.001 – 0.008
MVP	0.85	0.024	1.026	<2.23e-308	0.035 (0.002)	0.031 – 0.038
	0.90		1.258	<2.23e-308	0.035 (0.002)	0.031 – 0.038
	0.95		1.6	<2.23e-308	0.035 (0.002)	0.031 – 0.038
MGB	0.85	0.002	1.038	0.014	0.012 (0.006)	0.003 – 0.024
	0.90		1.264	0.014	0.012 (0.006)	0.003 – 0.024
	0.95		1.62	0.014	0.012 (0.006)	0.003 – 0.024
MSSM	0.85	-0.009	1.049	0.86	-0.016 (0.06)	-0.240 – 0.100
	0.90		1.331	0.868	-0.016 (0.069)	-0.242 – 0.118
	0.95		1.738	0.862	-0.016 (0.062)	-0.240 – 0.105
Meta-analysis	0.85	-	-	3.20E-70	0.025 (0.001)	0.022 – 0.208
	0.90	-	-	2.84E-70	0.025 (0.001)	0.022 – 0.208
	0.95	-	-	2.57E-70	0.025 (0.001)	0.022 – 0.208
Meta-analysis excluding MVP	0.85	-	-	0.066	0.005 (0.002)	-0.0003 – 0.009
	0.90	-	-	0.066	0.005 (0.002)	-0.0003– 0.009
	0.95	-	-	0.066	0.005 (0.002)	-0.0003– 0.009

© Sealock JM et al. *JAMA Psychiatry.*

eTable 13. Mediation Results With MDD Diagnosis as the Mediator Across PsycheMERGE Sites
mediated estimates were estimated using three different treatment percentiles of depression PGS (85%, 90%, and 95%) compared to the 50% percentile of PGS for controls.

**Cohort**	**MDD PGS Percentile**	**Control Percetile Value**	**Treatment Percentile Value**	**Proportion Mediated p-value**	**Proportion Mediated (SE)**	**Lower 95% CI**
VUMC	0.85	0.011	1.033	0.152	0.01 (0.011)	-0.004 – 0.032
	0.90		1.279	0.152	0.01 (0.011)	-0.004 – 0.032
	0.95		1.634	0.152	0.011 (0.011)	-0.004 – 0.032
MVP	0.85	0.024	1.026	<2.23e-308	0.162 (0.01)	0.143 – 0.181
	0.90		1.258	<2.23e-308	0.162 (0.009)	0.144 – 0.180
	0.95		1.6	<2.23e-308	0.162 (0.009)	0.145 – 0.180
MGB	0.85	0.002	1.038	0.012	0.044 (0.033)	0.011 – 0.108
	0.90		1.264	0.012	0.044 (0.033)	0.011 – 0.108
	0.95		1.62	0.012	0.045 (0.033)	0.011 – 0.109
MSSM	0.85	-0.009	1.049	0.784	-0.113 (0.57)	-1.409 – 1.003
	0.90		1.331	0.732	-0.104 (0.517)	-1.511 – 0.910
	0.95		1.738	0.73	-0.084 (0.56)	-1.042 – 1.014
Meta-analysis	0.85	-	-	5.91E-40	0.095 (0.007)	0.081 – 0.109
	0.90	-	-	1.78E-44	0.098 (0.007)	0.084 – 0.111
	0.95	-	-	9.73E-45	0.097 (0.007)	0.083 - 0.110
Meta-analysis excluding MVP	0.85	-	-	0.203	0.014 (0.011)	-0.007 – 0.035
	0.90	-	-	0.197	0.014 (0.011)	-0.007 – 0.034
	0.95	-	-	0.170	0.014 (0.010)	-0.006 – 0.034

© Sealock JM et al. *JAMA Psychiatry.*

eTable 14. Immune Subpopulation and Depression Diagnosis Mediation Analysis
Using a multiple mediator analysis, each subpopulation was modeled as the main mediator between the exposure and the outcome with the remaining subpopulations as alternative mediators.

**Outcome**	**Cell Type**	**Proportion Mediated**	**Lower 95% CI**	**Upper 95% CI**
MDD diagnosis	Basophils	0.005	-0.009	0.015
	Eosinophils	0.002	-0.015	0.014
	Lymphocytes	0.008	-0.007	0.018
	Monocytes	-0.001	-0.018	0.011
	Neutrophils	0.019	0.002	0.031

© Sealock JM et al. *JAMA Psychiatry.*

eTable 15. Results of Bidirectional Mendelian Randomization

**Exposure**	**Outcome**	b_{xy}	**SE**	**P-value**	**N SNPs**	**Multi SNP based HEIDI Outlier**
MDD	WBC	0.0223	0.0216	0.302	47	0.639
WBC	MDD	0.0272	0.0110	0.014	203	0.021

eTable 16. Results of Controlling for WBC Genetics Across PsycheMERGE Sites
Beta estimates were combined using a fixed-effects inverse weighted method.

**Outcome**	**Predictor**	**Cohort**	**PGS P-value**	**PGS Beta**	**PGS SE**	**PGS Lower 95% CI**	**PGS Upper 95% CI**
Depression diagnosis	WBC PGS	MSSM	0.915	0.008	0.073	-0.136	0.151
		MVP	4.71E-6	0.018	0.004	0.01	0.025
		MGB	0.856	-0.003	0.018	-0.039	0.032
		VUMC	0.57	-0.007	0.012	-0.03	0.017
		Meta-analysis	4.94E-5	0.015	0.004	0.008	0.022
WBC measurement	Depression PGS conditioned on WBC genetics	MSSM	0.934	0.003	0.041	-0.078	0.085
		MVP	2.26E-93	0.037	0.002	0.033	0.041
		MGB	1.26E-7	0.037	0.007	0.023	0.050
		VUMC	2.80E-12	0.025	0.004	0.018	0.031
		Meta-analysis	1.83E-28	0.027	0.002	0.022	0.032

© Sealock JM et al. *JAMA Psychiatry.*

eReferences

1. Gaziano, J. M. *et al.* Million Veteran Program: A mega-biobank to study genetic influences on health and disease. *J. Clin. Epidemiol.* **70**, 214–223 (2016).
2. Fang, H. *et al.* Harmonizing Genetic Ancestry and Self-identified Race/Ethnicity in Genome-wide Association Studies. *Am. J. Hum. Genet.* **105**, 763–772 (2019).
3. Karlson, E. W., Boutin, N. T., Hoffnagle, A. G. & Allen, N. L. Building the partners healthcare biobank at partners personalized medicine: Informed consent, return of research results, recruitment lessons and operational considerations. *J. Pers. Med.* **6**, 1–11 (2016).
4. Yang, J., Lee, S. H., Goddard, M. E. & Visscher, P. M. GCTA: A tool for genome-wide complex trait analysis. *Am. J. Hum. Genet.* **88**, 76–82 (2011).
5. Denny, J. C. *et al.* PheWAS: Demonstrating the feasibility of a phenome-wide scan to discover gene-disease associations. *Bioinformatics* **26**, 1205–1210 (2010).
6. Tingley, D., Yamamoto, T., Hirose, K., Keele, L. & Imai, K. Mediation: R package for causal mediation analysis. *J. Stat. Softw.* **59**, 1–38 (2014).
7. Bates, D., Mächler, M., Bolker, B. M. & Walker, S. C. Fitting linear mixed-effects models using lme4. *J. Stat. Softw.* **67**, (2015).
8. Beasley, T. M., Erickson, S. & Allison, D. B. Rank-based inverse normal transformations are increasingly used, but are they merited? *Behav. Genet.* (2009). doi:10.1007/s10519-009-9281-0
9. McCaw, Z. R., Lane, J. M., Saxena, R., Redline, S. & Lin, X. Operating characteristics of the rank-based inverse normal transformation for quantitative trait analysis in genome-wide association studies. *Biometrics* (2019). doi:10.1111/biom.13214
10. Astle, W. J. *et al.* The Allelic Landscape of Human Blood Cell Trait Variation and Links to Common Complex Disease. *Cell* **167**, 1415-1429.e19 (2016).
11. Zhu, Z. *et al.* Causal associations between risk factors and common diseases inferred from GWAS summary data. *Nat. Commun.* **9**, (2018).
12. Keller, M. F. *et al.* Trans-ethnic meta-analysis of white blood cell phenotypes. *Hum. Mol. Genet.* **23**, 6944–6960 (2014).

© Sealock JM et al. *JAMA Psychiatry.*
